# The role of cognition in nesting

**DOI:** 10.1098/rstb.2022.0142

**Published:** 2023-08-28

**Authors:** Topi K. Lehtonen, Heikki Helanterä, Cwyn Solvi, Bob B. M. Wong, Olli J. Loukola

**Affiliations:** ^1^ Ecology and Genetics Research Unit, University of Oulu, PO Box 3000, 90014 Oulu, Finland; ^2^ Guangdong-Hong Kong-Macao Greater Bay Area Center for Brain Science and Brain-Inspired Intelligence, Southern Medical University, Guangzhou 510515, China; ^3^ School of Biological Sciences, Monash University, Melbourne 3800, Victoria, Australia

**Keywords:** brain, ecological trap, environmental change, evolutionary framework, intelligence, parental care

## Abstract

For many animals, nests are essential for reproductive success. Nesting individuals need to carry out a range of potentially challenging tasks, from selecting an appropriate site and choosing suitable materials to constructing the nest and defending it against competitors, parasites and predators. Given the high fitness stakes involved, and the diverse impacts both the abiotic and social environment can have on nesting success, we might expect cognition to facilitate nesting efforts. This should be especially true under variable environmental conditions, including those changing due to anthropogenic impacts. Here, we review, across a wide range of taxa, evidence linking cognition to nesting behaviours, including selection of nesting sites and materials, nest construction, and nest defence. We also discuss how different cognitive abilities may increase an individual's nesting success. Finally, we highlight how combining experimental and comparative research can uncover the links between cognitive abilities, nesting behaviours and the evolutionary pathways that may have led to the associations between them. In so doing, the review highlights current knowledge gaps and provides suggestions for future research.

This article is part of the theme issue ‘The evolutionary ecology of nests: a cross-taxon approach’.

## 1. Introduction

**Figure 1.**
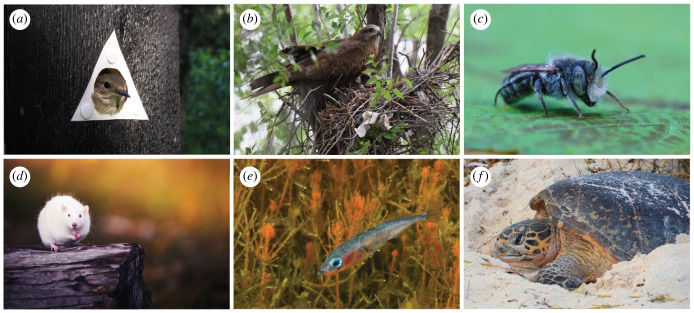
Putative evidence linking cognitive abilities with nesting behaviours is taxonomically widespread. Examples include (*a*) European pied flycatchers using perception, memory and learning when adjusting their nest site choices in response to the choices and performance of great tits; (*b*) black kites and (*c*) alfalfa leafcutting bees innovating through the use of anthropogenic materials to construct their nests; (*d*) rats learning from prior experience to improve their nest building; (*e*) sticklebacks showcasing precise perception and possibly prospective cognition when using the future risk of egg predation to adjust their nesting behaviours; and (*f*) hawksbill turtles having the cognitive abilities needed for deceiving would-be nest predators by engaging in sand scattering behaviour away from their nest. Photo credits: (*a*) Janne Seppänen, (*b*) Shutterstock/Nazin Alexandr, (*c*) Shutterstock/Wirestock Creators, (*d*) Shutterstock/Darina Matasova, (*e*) Mats Westerbom, (*f*) Shutterstock/Andrzej Grzegorczyk.

It has commonly been assumed that nesting behaviours are performed in a stereotypical and predictable manner, often without any prior experience or practice. For example, some bird parents retrieve an object close to their nest even if the object is not an egg, as long as it is similar enough to trigger the response [1]. Nest structures built by different individuals of some insect and bird species are so similar that they can be used in species identification [2,3]. Indeed, some nesting behaviours may not require memory, learning, problem solving or computation at all [4]. Even so, cognition may underlie improvement of such reactive behaviours over time, while other nesting behaviours performed by the same individual may require cognitive abilities from the start. For example, in the case of egg retrieval behaviour, the risk of brood parasitism may select for improved cognitive abilities that allow more fine-tuned assessment of objects near the nest, so that the nesting individual can more flexibly recognize and retrieve its own egg while rejecting other similar objects [5]. Here, we define cognition as the mechanism by which animals acquire, process and store information to act upon it [6,7]. We can therefore expect it to be associated with actions that are above and beyond simple reactive behaviours. Moreover, we consider nests as structures that hold eggs, young, or both, and we focus on the behaviours needed in nesting site and material selection, nest construction and nest defence. We discuss examples from a wide range of taxa (figure 1) that link cognitive abilities to nesting behaviours. Many of these studies were not carried out from a cognitive perspective, and we therefore offer potential interpretations. With this approach, we aim to highlight current knowledge gaps to invigorate new research towards addressing those gaps and to broaden our understanding of the evolutionary processes that link cognition with nesting behaviours.

## 2. Evidence linking cognitive capacities and nesting behaviours

### (a) Nest site selection

Learning can play an important role in nest site selection (and other nesting behaviours). In common eiders (*Somateria mollissima*), the ability to use prior experiences to choose a safer site in future nesting attempts most likely explains the improved nesting success of older females [8]. Some nesting animals also seem to act on cues about the future state of the world, implying prospective cognition, i.e. having an understanding of the outcome of current actions [9,10]. For instance, monk parakeets (*Myiopsitta monachus*) decide where to build a nest based on the local environment's topographical qualities, preferring to nest on electric towers with more angular structures, which probably provide better support for the completed nest. Their preferred nesting spots also have higher structures nearby, which can later serve as perches. Finally, parakeets prefer to have paved pathways near their nest sites, putatively because these provide shallow pools of water after rain [11]. Similarly, cliff swallows (*Petrochelidon pyrrhonota*) choose nest locations that are closer to nesting materials and sites that allow building of less time-consuming nests, thus suggesting that these birds are making initial decisions based on minimizing the time and energy they later need to invest in nest construction [12].

Nests and nesting sites can also function as cues that provide inadvertent information to others about, for example, the location, mating decisions, social position, dominance, body condition and cognitive capabilities of the nest owners [13]. Cognitive abilities, such as social learning, memory, perception and attention, are likely to play an important role in the use of social cues in the context of nesting decisions, both within [14,15] and between [16] species. As an example of the former, many birds, such as red-winged blackbirds (*Agelaius phoeniceus*) [17], house wrens (*Troglodytes aedon*) [18], collared flycatchers (*Ficedula albicollis*) [19] and great tits (*Parus major*) [20], use the current or old nests of conspecifics as cues when assessing the quality of a potential nest site. Goldeneye duck (*Bucephala clangula*) females that parasitise nests of conspecifics prefer to lay eggs in host nests that were successful in the previous year, while avoiding unsuccessful nests, based on the parasitic female's nest prospecting in the preceding season [21]. The ability to use such fine-tuned nest-site selection criteria requires memory, perception and decision-making capacities over the time scale of years and is therefore likely to represent cognitive abilities beyond simple associative learning. In a heterospecific context, European pied flycatchers (*Ficedula hypoleuca*) (figure 1*a*) are sensitive to the nest-site choices and performance of tits (*Parus* spp.), for example by copying nest-site features of the tits they have observed to have large clutches, while rejecting those associated with small clutches [22–24]. Similarly, female blue mason bees (*Osmia caerulescens*) and orange-vented mason bees (*O*. *leaiana*) either copy or reject nest site features of red mason bees (*O*. *bicornis*), depending on whether the nesting attempts were successful [25]. Such an active selection strategy to selectively copy nest-site features of other species, based on their observed success, may be common in animals that share similar nesting requirements and habitats [24,25]. The associated cognitive abilities may seem complex but can be achieved with very small brains and explained by associative learning mechanisms (*sensu* [26–29]).

### (b) Nesting material choice

Learning can also allow nesting individuals to improve their nesting material selection. In zebra finches (*Taeniopygia guttata*), previous experience was found to influence nesting material choice: birds learned to prefer stiffer strings as a nest building material, thereby using fewer strings for a nest and increasing their nest building efficiency [30]. Hence, the birds seem to learn to choose suitable nest material based on its suitability for a physical task, implying sophisticated ‘physical cognition’ [30,31]. Zebra finches also learn to improve with experience when choosing nesting material based on the size of their nest's entrance hole [32]. Moreover, male zebra finches changed their nesting material preference based on whether the material was associated with egg removal during the previous nesting attempt [33]. Besides the demonstrated role of learning in nesting material choice, some of the best-known examples of innovation, the ability to come up with new solutions to old problems [34], come from the exploitation of novel materials in nest construction. In order to innovate, animals must inhibit their previously learned or innate responses in favour of new solutions and, for this to occur, they may need to have good problem-solving abilities [35] and overcome the tendency for risk aversion and neophobia [36]. Indeed, a comparison over hundreds of bird species links innovation ability and cognition [37]. Notably, innovation can be especially advantageous in challenging or changing environmental conditions [38]. The use of novel materials by certain bird species has been increasing over time and is especially common in human-modified environments, such as cities and farmland [39,40]. For example, black kites (*Milvus migrans*) (figure 1*b*) living in cities have taken to adorning their nests with white plastic materials that can convey reliable information to conspecifics about the viability, territory quality and fighting ability of the nest builder [41]. While the innovative use of anthropogenic nesting materials appears to be taxonomically widespread, recent research demonstrates interesting phylogenetic biases. For example, a study of birds across the Australian continent found that the three avian families with the highest incidences of anthropogenic nesting material use were also well-known urban exploiters, biological invaders, or both [40].

The ability to innovate via the use of anthropogenic nesting materials can confer either positive or negative fitness consequences. Regarding the former, synthetic materials, such as plastic string, may help to reinforce the structure of the nest or provide insulation, as in great grey shrikes (*Lanius excubitor*) [42]. As a result, the use of such materials has the potential to enhance offspring survival by providing much needed protection to eggs and nestlings from adverse weather conditions [42]. Some anthropogenic nesting materials have also been shown to repel or suppress the proliferation of nest parasites, which can otherwise compromise nestling survival [43]. For instance, the use of discarded cigarette butts as nesting material is common in urban-dwelling birds, with the toxicants present in smoked cigarettes acting as powerful deterrents against ectoparasites [44,45]. Similarly, alfalfa leafcutting bees (*Megachile rotundata*) (figure 1*c*) may impede parasitic infection by using anthropogenic materials in nest construction, with materials such as plastics providing particularly effective defence barriers against infiltration by host-seeking parasitoids [46]. However, such a shift to the use of novel material may also have adverse fitness outcomes. For example, when leafcutting bees construct brood cells inside plastic straws, the developing young may experience increased mortality due to mould that thrives because of the poor moisture diffusing properties of the straws [46]. Similarly, strings incorporated into the nests of great grey shrikes may cause entanglement-related injury and death of both offspring and adults [42].

### (c) Nest construction and nest architecture

Cognition can also play a key role in nest construction and nest architecture. Laboratory rats (*Rattus norvegicus domestica*) (figure 1*d*), for example, built more elaborate nests as adults if allowed to interact with nesting material as juveniles, implying that learning from prior experience allowed the rats to improve their nest building [47]. Similarly, young village weaverbirds (*Ploceus cucullatus*) first build crude, loose structures for nests, whereas older individuals construct much more neatly woven, compact and organized nests, presumably reflecting learned weaving skills [48]. Intuitively, the ability to flexibly adjust nest construction to varying, and sometimes conflicting, demands of the physical and social environment can be expected to involve cognition, but we need future studies to assess to what extent this is really the case. For instance, it is not known what abilities (if any) male common gobies (*Pomatoschistus microps*) use to tune their nest construction (and tending) in response to the conflicting demands of egg ventilation and predation risk [49], or how sand gobies (*Pomatoschistus minutus*) factor in their body size when adjusting nest architecture to salinity, social environment or egg predation risk [50–52].

Nest construction of some arthropods has been suggested to involve a level of prospective cognition [9]. Honeybees (*Apis mellifera*) are able to adjust the size and structure of their comb cells to merge separate sections, cope with irregular foundations and generate curved architecture to avoid obstacles [53]. Allegedly, these nest building behaviours are best explained by a combination of reactive behaviours and cognitive abilities that allow a basic understanding of the overall desired outcome [9,54]. However, whether any future thinking is involved, or whether simpler heuristics could account for these behaviours, will require further experimental investigation.

Sometimes nest construction and completed nests function as ‘extended phenotype signals’, which can reveal important information about the nest builder, from fighting prowess [41] to cognitive abilities [55]. In many taxa this social information is, in turn, used in reproductive decisions, including mate choice [55–58]. In the context of nests, the use of social information may select for cognitive abilities—such as enhanced perception, memory, and learning abilities—especially if the fitness of both the sender and receiver is affected. The situation may even promote the coevolution of cognitive abilities of nest owners and information users. For example, the capacity of European pied flycatchers to eavesdrop on both the clutch size and nest structure of great tits has implications for both species. By deciphering the information to selectively copy nest-site characteristics, flycatchers may increase their nesting success, while niche convergence [24] and decreased number and condition of great tit fledglings may also follow [59]. If tits evolve counter-adaptations, an evolutionary arms race [60] between the two species in acquiring and hiding nest-related information results [16,61], which could create a selection regime for increased performance in the above-mentioned cognitive abilities that facilitate efficient social information use. In the specific case of these two species, however, opportunities for such an arms race could also depend on a suite of environmental factors, such as climate change [62], highlighting the complexity of the interactions that can ensue.

### (d) Nest defence

The ability to flexibly respond to environmental conditions may suggest underlying cognition also in the context of nest defence. In three-spined sticklebacks (*Gasterosteus aculeatus*) (figure 1*e*), males seem to consider the expected future risk of egg predation when adjusting some of their nesting behaviours. Specifically, in the presence of an egg predating shrimp (*Palaemon elegans*), male sticklebacks were less likely to initiate nest building and invested less in both egg fanning and territory defence [63]. One strategy by which animals can reduce the incidence of predation on eggs and young is to conceal, disguise or camouflage their nest [64,65]. For instance, some species, such as hawksbill (*Eretmochelys imbricata*) (figure 1*f*) and leatherback (*Dermochelys coriacea*) turtles, scatter sand away from their nest to create decoy trails to deliberately misguide would-be egg predators as to the location of the actual nest [66]. Nest builders may also have the capacity to choose nesting materials (see §2b, above) so that it helps with camouflage. Zebra finch males, for example, chose to nest mostly with material that matched the colour of the nest cup and surrounding cage walls, hence actively selecting materials that helped camouflage their nests [67]. While we suggest that precise memory, perception, and even prospective cognition would be beneficial when engaging in such nest concealment behaviours, more work is needed to determine the range of cognitive abilities that may be involved.

The mimicry–recognition arms race between brood parasites and their hosts reveals a link between cognitive abilities and evolutionary dynamics [68], with the defence responses of hosts being pushed to overcome the deception by the parasites [69,70]. The need to expel eggs (or later chicks) of heterospecific or conspecific nest parasites has selected for sophisticated host defences, including the use of multiple sources of information in the decision process (indicating a cognitive capacity for complex decision making [71]) and counting the number of eggs laid (indicating a level of numerical cognition [72]). For instance, while cuckoos impose high reproductive costs on their hosts when their hatchling evicts the host's young from the nest [73], not all host individuals are able to reject the parasitic egg(s), implying variation in their cognitive abilities related to perception and decision making [68]. When using a similar parasitic strategy, the cuckoo catfish (*Synodontis multipunctatus*) learns to overcome host defences during the parasite's lifetime; a cognitive feat that may also be displayed by other brood parasites [74]. Interestingly, some ants, social wasps and social bees also employ cuckoo-like strategies to parasitize heterospecific nests, with both parasites [75,76] and hosts [77] having evolved sophisticated strategies of deception or defence.

## 3. Future directions

### (a) Evolutionary framework

We have highlighted established and potential examples of cognition underlying different nesting behaviours across a wide range of ecological settings. In this section, we outline how to apply an established evolutionary framework for addressing the role of cognition in nesting. Here, a robust evolutionary understanding is built by integrating three approaches: conducting experiments that demonstrate the cognitive abilities involved, collecting data on how natural selection shapes such abilities, and performing comparative analyses that address their evolutionary history and patterns across taxa. This framework can help, not only in bridging taxonomic divides and testing novel predictions, but also in gaining an understanding of the significance of cognition in nesting behaviours in a world of rapid and unprecedented anthropogenic change. Below, we discuss the utility of each of the framework's three components.

First, controlled experiments are essential for robustly demonstrating the use of cognition in nesting behaviours. In particular, such experiments, carried out in the laboratory or field, can uncover patterns that are otherwise elusive in the wild, if the environmental setting does not vary in a desired manner. Here, experiments that, for example, manipulate likely fitness consequences of nesting decisions [50,52,78], manipulate information availability [13], or expose nesting individuals to novel environmental conditions [79] are important for understanding the cognitive processes underlying nesting decisions and also give insight into their potential adaptive value.

Second, the adaptive hypotheses emerging from experiments need to be complemented by analyses of fitness consequences of, and natural selection on, cognitive abilities in the wild. Ideally, such analyses will reveal whether fitness differences between individuals arise from variation in cognitive abilities *per se*, rather than from covariates of cognitive performance, such as differences in personality or body condition (e.g. nutritional status or parasite load) [80]. Furthermore, given the possibility that the adaptive value of a given cognitive ability only occurs under certain conditions [81], we must understand the range of conditions under which the fitness benefits occur and the prevalence of those conditions in the wild. For example, during environmentally challenging breeding seasons, female common eiders with bigger brains (a contentious proxy of cognition but presumably relevant in eiders [82]) attained higher egg hatching success (proxy of fitness) and were more successful in forming antipredator brood-rearing coalitions, whereas, in more benign years, females that had invested less in brain size may have an advantage [82,83].

Measures of cognitive performance have been shown to correlate with fitness proxies in wild populations of both vertebrates and invertebrates, and to respond to selection in laboratory populations [80]. Inferring selection on cognitive traits in the wild is, however, challenging, because individuals may trade off different fitness components against each other. For example, although great tit females with particularly good problem-solving skills laid more eggs, such females were also more likely to abandon their nests and produce no fledglings, resulting in no overall selective benefits being observed [84]. Thus, it remains to be shown whether, and under what conditions, the use of cognitive abilities in a nesting context drives fitness variation and responds to selection in the wild. This would be the ultimate demonstration of the adaptive value of cognitive abilities in nesting.

Third, to complement experimental data, we need phylogenetic comparative analyses [85] that map occurrence of a trait across species into a phylogeny and hence give insights into its evolutionary history. By revealing the correlates of trait variation across species, such analyses may provide support for adaptive hypotheses. This approach has, for example, demonstrated the link between nest size and environmental features in birds [86] and that cooperative breeding facilitates colonization of harsh environments [87]. Comparing whether, within a phylogeny, the use of cognitive abilities in nesting co-occurs with a specific trait or feature can be a powerful way of demonstrating evolutionary correlates of cognitive abilities. Such correlates can include environmental conditions (e.g. stability, harshness or complexity of the environment), life-history (life-span, semelparity versus iteroparity) or social traits of the species (cooperative breeding, group size, mating system). The correlates of cognitive abilities also include physiological aspects, such as the underlying neural circuitry [88,89], or the energetic constraints on investment into neural tissue [90]. An understanding of how such proximate factors facilitate or constrain the evolution of cognitive abilities helps to unravel the ecological conditions under which cognition plays an important role in nesting and whether similar mechanisms are shared across taxa.

A particularly illuminating comparative investigation would be to study whether cognitively complex nesting behaviours predispose a taxon to evolving towards the use of the associated cognitive skills in other contexts. Similarly, the cognitive skills currently used in nesting may have first evolved in other contexts and later been co-opted for nesting purposes. The evolution of cognitive abilities is often argued to be driven by social complexity and the challenges of food acquisition in complex environments (as per the social brain and ecological intelligence hypotheses [91,92]). Given the sophistication and presumably high fitness significance of nesting behaviours, it is relevant to ask whether they could be another key driver of the evolution of cognitive abilities. Mapping the phylogenetic distribution of different measures of cognitive performance across multiple contexts could allow researchers to tease apart whether nesting is associated with certain cognitive skills particularly often, or whether it is more likely that cognitive skills applied in nesting have primarily evolved in other contexts. For example, it has been argued that tool use and nest construction may require similar skills as those needed in object manipulation and material choice [93], raising questions about the context in which the skills might have originally evolved. Similarly, cognitive skills, such as spatial memory, social learning and anticipation of future conditions, may have been co-opted across contexts [94], with each being applicable in nesting, foraging and social contexts. Here, phylogenetic comparative methods are highly suitable for studying the context in which cognitive skills were likely to originally evolve, and what their evolutionary consequences may have been.

Experimental, field and comparative approaches intertwine: meaningful comparative analyses depend on unbiased data, and given that the aim is to test hypotheses on traits that require experimental manipulations or field monitoring, acquiring the data is not trivial and could involve considerable effort. Future field and experimental work should also be wary of taxonomic biases in the choice of study organisms for investigating particular cognitive abilities. Furthermore, for meaningful analyses and a robust synthesis, we need to be cognisant of publication biases caused by the underreporting of studies in which no link between cognition and nesting behaviours is found.

### (b) Cognitive abilities in a changing world

Another important avenue for future research is to understand the role of cognition in animals' responses to changes in environmental conditions, especially with regard to those of anthropogenic origin. Here, we should note that even when nesting individuals are equipped with appropriate cognitive abilities, responses to the environment are not always possible and, even when they are, may not necessarily be adaptive [38,95]. For example, in Crater Lake Apoyo, parents of the critically endangered arrow cichlid (*Amphilophus zaliosus*), evolutionarily naive to the dangers posed by a non-native brood predator, the bigmouth sleeper (*Gobiomorus dormitor*), appear incapable of recognizing or learning to associate the severity of the threat that is posed by the novel predator. This inability allows the sleeper to venture perilously close to the offspring before the parents mount an appropriate antipredator response [96]. Similarly, nesting animals habituated to humans may, as a result of stimulus generalization, forego appropriate responses to native or introduced (nest) predators [97]. Novel conditions associated with human-mediated environmental change can also result in ‘ecological traps’, which can occur when altered conditions lead to a mismatch between a habitat’s actual quality and the cues that individuals process when assessing the habitat [98–100]. For example, great tits that choose to use nest boxes installed in forest patches afflicted by outbreaks of the tits' food source, the great web-spinning sawfly (*Acantholyda posticalis*), ultimately experience reduced fledgling success and poorer fledgling condition due to decreased resource availability caused by the vegetation damage inflicted by the ravenous sawfly larvae [101]. Lastly, even when cognitive skills in the context of nesting are adaptive, they may not always be sufficient to counter the impacts of anthropogenic change. It is therefore important to consider whether high cognitive performance in nesting behaviours increases nesting success and, if it does, whether this ability is sufficient, adaptive and adequate in countering the impacts of anthropogenic change [38].

## 4. Conclusion

In this review, we discussed, across taxa, empirical examples of how cognitive abilities can benefit nesting behaviours in much the same way as in other fitness-related activities, such as building non-nest structures (e.g. for shelter), engaging in social interactions and finding food. In particular, we focused on the putative roles of cognitive abilities in nest site selection, nesting material choice, nest construction, and nest defence. In doing so, we uncovered how few studies have explicitly linked cognitive abilities with nesting behaviours. Accordingly, we argued that to improve our understanding of cognitive abilities in various nesting contexts, we need assessments of their adaptive value and evolutionary history by integrating experimental approaches, conducting selection studies in natural populations, and using broad comparative settings. Finally, we identified human-induced environmental impacts as a major research opportunity to increase our knowledge of both the role of cognition in nesting and the scope of nesting animals to adapt to a world of rapid and unprecedented change. We hope that our synthesis will invigorate new research into the links between cognition and nesting behaviours.

## Data Availability

This article has no additional data.
